# Poor Outcomes in a Cohort of HIV-Infected Adolescents Undergoing Treatment for Multidrug-Resistant Tuberculosis in Mumbai, India

**DOI:** 10.1371/journal.pone.0068869

**Published:** 2013-07-19

**Authors:** Petros Isaakidis, Roma Paryani, Samsuddin Khan, Homa Mansoor, Mamta Manglani, Asmaa Valiyakath, Peter Saranchuk, Jennifer Furin

**Affiliations:** 1 Médecins Sans Frontières, Mumbai, India; 2 Pediatric Centre of Excellence for HIV Care, Lokmanya Tilak Municipal Medical College and General Hospital, Sion, Mumbai, India; 3 Southern Africa Medical Unit, Médecins Sans Frontières, Cape Town, South Africa; 4 Tuberculosis Research Unit, Case Western Reserve University, Cleveland, Ohio, United States of America; Institute of Infectious Diseases and Molecular Medicine, South Africa

## Abstract

**Background:**

Little is known about the treatment of multidrug-resistant tuberculosis (MDR-TB) in HIV-co-infected adolescents. This study aimed to present the intermediate outcomes of HIV-infected adolescents aged 10–19 years receiving second-line anti-TB treatment in a Médecins Sans Frontières (MSF) project in Mumbai, India.

**Methods:**

A retrospective review of medical records of 11 adolescents enrolled between July 2007 and January 2013 was undertaken. Patients were initiated on either empirical or individualized second-line ambulatory anti-TB treatment under direct observation.

**Results:**

The median age was 16 (IQR 14–18) years and 54% were female. Five (46%) adolescents had pulmonary TB (PTB), two (18%) extrapulmonary disease (EPTB) and four (36%) had both. Median CD4 count at the time of MDR-TB diagnosis was 162.7 cells/µl (IQR: 84.8–250.5). By January 2013, eight patients had final and 3 had interim outcomes. Favourable results were seen in four (36.5%) patients: one was cured and three were still on treatment with negative culture results. Seven patients (64%) had poor outcomes: four (36.5%) died and three (27%) defaulted. Three of the patients who died never started on antiretroviral and/or TB treatment and one died 16 days after treatment initiation. Two of the defaulted died soon after default. All patients (100%) on-treatment experienced adverse events (AEs): two required permanent discontinuation of the culprit drug and two were hospitalized due to AEs. No patient required permanent discontinuation of the entire second-line TB or antiretroviral regimens.

**Conclusions:**

Early mortality and mortality after default were the most common reasons for poor outcomes in this study. Early mortality suggests the need for rapid diagnosis and prompt treatment initiation, and adolescents might benefit from active contact-tracing and immediate referral. Default occurred at different times, suggesting the need for continuous, intensified and individualized psychosocial support for co-infected adolescents. Operational research among co-infected adolescents will be especially important in designing effective interventions for this vulnerable group.

## Introduction

Multidrug-resistant tuberculosis (MDR-TB)–defined as strains of TB with *in vitro* resistance to at least isoniazid and rifampin–is a major public health problem [1]. In 2010, it was estimated that there were 650,000 prevalent MDR-TB cases, few of which were actually diagnosed and treated. In fact, fewer than 40,000 patients have been put on World Health Organization (WHO)-recommended therapy in the last decade [2]. Inadequate diagnosis and treatment of MDR-TB is even worse in children, who represent an estimated 10–20% of all cases, up to 80,000 each year [3]–[4]. The published literature reports only a small number of pediatric patients receiving treatment, and a recent meta-analysis of pediatric MDR-TB treatment outcomes included only 315 children [5].

What little pediatric data does exist tends to group all outcomes together, despite the fact that the data represents children as young as a few months old and others up to 18 years of age [6]. It is widely acknowledged that younger children face more challenges in terms of diagnosis and medication dosing [7] while older children, especially those in what is known as the “adolescent” age group (defined by the World Health Organization as those aged 10–19 years [8]), may face more challenges with adherence given their developmental state [9]. The literature on chronic disease management in adolescents has shown that this population has special physical and psychological needs [10]–[12]. Adolescents often experience spurts of growth that may lead to under-dosing with their medication [13]. Certain diseases, including TB, may also present more aggressively in this population [14]–[15]. Perhaps more significantly, adolescence is defined as a period of emotional and psychological upheaval that can affect relationships with health care providers and caregivers and ultimately adherence to medical regimens [16]–[17]. In addition, adolescence is a time period during which children must transition into adult roles; there may be increased time constraints due to school, work or family responsibilities [18]–[20]. All of these issues can affect the health outcomes of adolescent populations with chronic diseases, such as MDR-TB.

To date, there are no published reports characterizing MDR-TB treatment outcomes in the adolescent population. This paper fills that gap by presenting data from a cohort of 11 adolescent patients diagnosed with MDR-TB in Mumbai, India, all of whom were co-infected with HIV. The cohort is relatively small, but is being reported here because it demonstrates some concerning findings in the adolescent population undergoing treatment for MDR-TB. Targeted interventions to improve MDR-TB treatment outcomes in adolescents are also discussed.

## Methods

### Study Design, Setting and Study Population

This is a retrospective review of the medical records of HIV-infected adolescents aged 10–19 years with culture-confirmed or suspected MDR-TB, undertaken as part of a larger study on MDR-TB treatment outcomes in Mumbai, India [21]. All patients were enrolled to receive treatment with second-line anti-TB medications in a Médecins Sans Frontières (MSF) clinic. Patients were referred to the clinic by public antiretroviral treatment centers (ART Centers), including the Regional Pediatric ART Centre, L.T.M. Medical College, Sion, Mumbai, which serves as one of the Centres Of Excellence in Pediatric HIV Care in India. Patients were also referred to the clinic by a network of community non-governmental organizations. Eleven (11) adolescents enrolled in care between July 2007 and January 2013 are included in this analysis.

### Treatment Protocol and Follow-up

The ambulatory, community-based MDR-TB treatment program has been described in detail in a previous publication [21]. Individualized treatment regimens are designed for each patient, based on drug susceptibility testing (DST) and prior treatment history. Standardized empiric treatment is given to patients who require immediate initiation due to disease severity or for those in whom a DST result is not available but in whom MDR-TB is diagnosed based on clinical findings, past TB treatment history and/or history of contact with a known or suspected MDR-TB patient. The standardized regimen follows international recommendations [22] and includes six drugs: pyrazinamide, capreomycin, moxifloxacin, ethionamide, cycloserine and p-aminosalicylic acid (PAS) and is modified as necessary if DST results become available. Blood test monitoring was done every month and sputum smear and culture every month during the intensive phase and every three months during the continuation phase. Treatment is continued for a minimum total duration of 18–20 months, including a minimum of 6–8 months intensive phase that includes an injectable agent. Antiretroviral therapy (ART) against HIV includes two nucleoside/tide reverse transcriptase inhibitors (NRTIs) and one non-nucleoside reverse transcriptase inhibitor (NNRTI) in a first-line ART regimen, while patients in need of second-line ART receive a protease inhibitor-based regimen along with NRTIs that are likely to be effective, based on HIV resistance testing results.

The adolescent patients were evaluated and followed by a multidisciplinary team of doctors, nurses, a psychologist and a social worker. They were clinically evaluated every two weeks during the first month of treatment and once a month thereafter. Home visits were conducted by the clinic team as necessary in order to provide social and emotional support to the adolescents and their families. At the community level, a network of public and private health structures and non-governmental organizations (NGOs) acted as providers of directly observed therapy (DOT) for the MDR-TB medication. DOT providers were given training on adverse events, adherence monitoring and pill counts, and referral pathways for patients with poor adherence.

### Data Collection and Analysis

Demographic and clinical information were systematically recorded in clinical files and entered into an electronic database. Information on HIV and antiretroviral therapy was collected in the same patient file but entered in a separate database. Each patient had a unique identification code that was used in both databases. Possible outcomes, defined according to WHO guidelines, included: cure, treatment completed, death, default, transfer out, treatment failure, and ‘alive and on treatment’.

### Ethics

This study has met the criteria for analysis of routinely collected program data of the MSF Ethics Review Board, Geneva, Switzerland. As this was a study of routinely collected monitoring data, informed consent from the patients was not obtained. The named ethics committee waived the need for consent.

## Results

Eleven adolescents, aged 10 to 19 years, diagnosed with MDR-TB and co-infected with HIV are reported here. All had perinatally acquired HIV. The median age was 16 [Interquartile Range (IQR): 14–18] and 54 percent were female. All except one had a past history of TB treatment and four of them were contacts of known drug-resistant TB patients. Five (46%) adolescents had pulmonary TB, two (18%) had extrapulmonary disease and four (36%) had both pulmonary and extrapulmonary TB (Table 1).

**Table 1:** Demographic and Clinical Characteristics of the Mumbai HIV/MDR-TB co-infected adolescent cohort, 2007–2013 (N = 11).

Characteristic	n (%)
Age, median (IQR) years	16 (14–18)
Female gender	6 (54)
Site of disease	
Pulmonary	5 (46)
Extrapulmonary	2 (18)
Pulmonary+Extrapulmonary	4 (36)
Previous TB treatment	10/11 (91)
MDR-TB Contact	4/11 (37)
Resistance (when DST available)	
H	9/9 (100)
R	9/9 (100)
E	7/9 (78)
FQ	6/8 (75)
Injectable	1/8 (13)
CD4 count at time of MDR-TB diagnosis, cells/µl, median (IQR)	162.7 (84.8–250.5)

IQR: interquartile range; H: isoniazid; R: rifampicin; E: ethambutol; FQ: fluoroquinolones.

**Figure 1:**
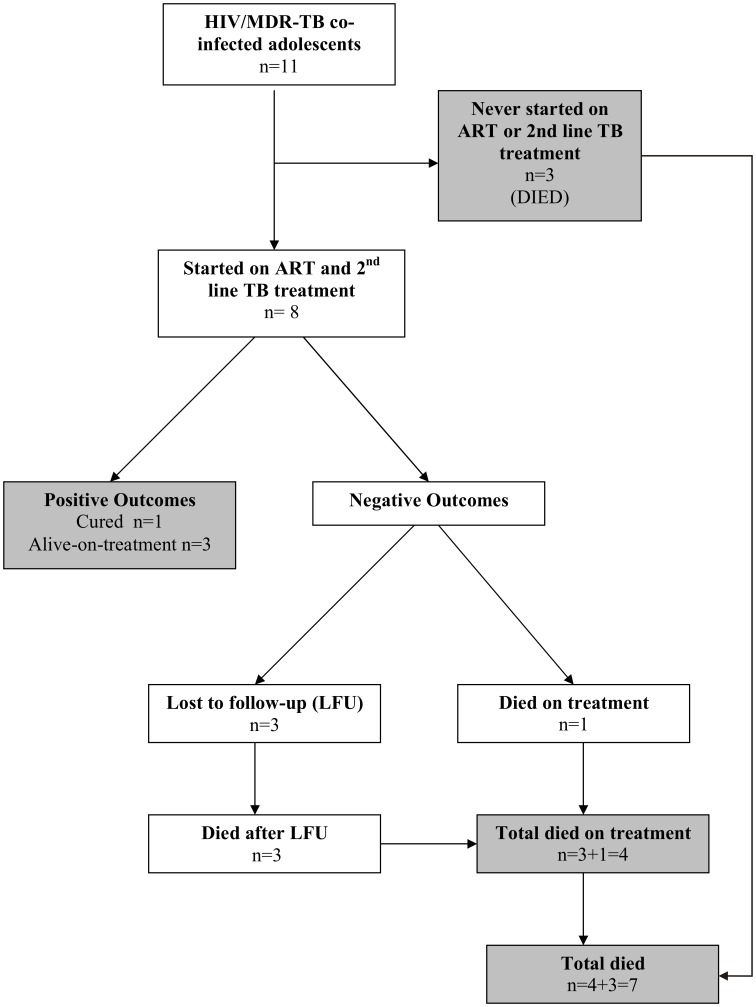
Flowchart of the Mumbai HIV/MDR-TB co-infected adolescent cohort, 2007–2013.

The median CD4 count at the time of MDR-TB diagnosis was 162.7 cells/µl (IQR: 84.8–250.5). Five patients were on ART at the time of the MDR-TB diagnosis: two of them were on ART for more than 2 years and three of them for six months or less. Another three patients were started on ART following initiation of DR-TB treatment: two patients after seven months and one patient after one month of TB treatment (Table 2). The median time from diagnosis of MDR-TB to initiation of second-line treatment was 10 days (IQR: 7–12). Three patients did not start antiretroviral and second-line TB treatments as they died soon after enrolment (Figure 1). Routine HIV viral load monitoring was not available during the early years of the program.

**Table 2:** Clinical Characteristics of the Mumbai HIV/MDR-TB co-infected adolescent cohort, 2007–2013.

	Age/Sex	CD4*	ART Regimen	ART Started	TB Site	Smear	Culture	Resistance	DR Type	2^nd^-Line TB Regimen
1	14/M	243	D4T+3TC+EFV	5 months prior	P+EP (Abd LN)	NEG	POS	S,H,R,E,Km,PAS,Ofx,Eto	XDR	Cm,Lfx,Eto,Cs,Amcl
2	10/M	273	D4T+3TC+NVP	7 months after	P	NEG	POS	S,H,R,E,Ofx,Eto	Pre-XDR	Km,Mfx,Eto,Cs,PAS,Z,Amcl
3	18/F	125	TDF+3TC+EFV	5 months prior	P	POS	POS	S,H,R,E,Ofx	Pre-XDR	Km,Mfx,Eto,Cs,PAS
4	13/F	147	No ART	Never	EP (LN)	POS	POS	S,H,R,E,Ofx	Pre-XDR	
5	18/F		No ART	Never	P	POS	POS	S,H,R,E	MDR	
6	16/F	92	D4T+3TC+EFV	6 months prior	P+EP (Cereb)	POS	POS	S,H,R,E,PAS,Z	MDR	Cm,Mfx,Eto,PAS,Z,H,Amcl
7	15/F	199	AZT+3TC+NVP	7 months after	P	POS	POS	S,H,R,E,PAS,Z	MDR	Km,Mfx,Eto,Cs,Amcl,H,E
8	19/F	290	TDF+3TC+ATV/r	28 months prior	P	NEG	POS	S,H,R,E,Ofx,PAS	Pre-XDR	Cm,Mfx,Eto,Cs,H,Cfz,Z
9	14/M	159	ABC+3TC+LPV/r	71 months prior	EP Abd	NEG	NEG	_	Empirical	Cm,Mfx,Eto,Cs,PAS,Z
10	16/M	36	No ART	Never	P+EP (Abd LN)	NEG	NEG	_	Empirical	
11	18/M	63	TDF+3TC+EFV	1 month after	P+EP (Abd)	NEG	POS	S,H,R,E,Ofx,PAS,Mfx,Z	Pre-XDR	Cm,Lfx,Eto,Cs,Amcl,Hh,Cfz

M: male; F: female; D4T: stavudine; 3TC: lamivudine; EFV: efavirenz; TDF: tenofovir; AZT: zidovudine; ABC: abacavir; NVP: nevirapine; ATV/r: atazanavir/ritonavir; LPV: lopinavir; PTB: pulmonary tuberculosis; EPTB: extra-pulmonary tuberculosis; Abd; abdominal; Cereb; cerebral, LN: lymph node; NEG: negative; POS: positive; S: streptomycin; Cm: capreomycin; Km: kanamycin; Lfx: levofloxacin; Eto: ethionamide; CS: cycloserine; Amcl: amoxicillin/clavulanic acid; Mfx: moxifloxacin; Ofx; ofloxacin; PAS; para-aminosalicylic acid; Z: pyrazinamide; H: isoniazid; Hh: high dose isoniazid; E: ethambutol; Cfz: clofazimine; XDR: extensively drug-resistant; MDR: multidrug-resistant.

* at time of MDR-TB diagnosis.

By January 2013, eight patients had final outcomes and three had interim outcomes (Table 3). Favourable outcomes were seen in 4 (36%) of the patients, one of whom was cured and three of whom were still on treatment with negative smears and/or cultures and improving clinical signs. Two of these latter patients were about to complete their course of second-line anti-TB treatment at the time of this analysis and they were doing very well.

**Table 3:** Outcomes of the Mumbai HIV/MDR-TB co-infected adolescent cohort, 2007–2013.

No	Age/Sex	MDR Tx Start Date	Outcome	Time on Treatment	Reason for default/Cause of death/Comments
1	14/M	27.07.2007	**Defaulted**	17 Months	Patient defaulted after 17 months of treatment and then died 1 month after default. Convulsions, Brain Tuberculoma.
2	10/M	31.08.2007	**Cured**	18 Months	
3	18/F	19.06.2008	**Defaulted**	3 Months	Patient died 1.5 months after default. Reason for default: adverse events & social issues. Cause of death not ascertained.
4	13/F	Never	**Died**	0 Months	Died before treatment, 1 month after enrolment
5	18/F	Never	**Died**	0 Months	Patient declined treatment and died 2 months later
6	16/F	15.02.2011	**Died**	16 Days	Brain Tuberculoma
7	15/F	22.02.2011	**Defaulted**	21 Months	Patient defaulted a few weeks before treatment completion due to social issues (family deaths, sexual abuse) & personal issues (sexual debut, not willing to disclose status to partner)
8	19/F	12.05.2011	**Alive on Treatment**	21 Months	Patient has culture converted, about to complete treatment
9	14/M	24.11.2011	**Alive on Treatment**	15 Months	Patient in excellent clinical condition
10	16/M	Never	**Died**	0 Months	Died before treatment, 1 month after enrolment. Acute renal failure.
11	18/M	23.10.2012	**Alive on Treatment**	4 Months	Adherence problems (behavioral issues, adverse events)

The remaining 7 patients (64%) had poor treatment outcomes: four of these patients (36.5%) died and three of them (27%) defaulted treatment. Of the four who died, three never started on MDR-TB and ART treatments and one died 16 days after treatment initiation. Treatment interruptions were recorded in all three patients who defaulted treatment. Two of the adolescents who defaulted died soon after they abandoned treatment at 3 and 17 months, reportedly due to adverse events and social problems respectively. One patient, a 15 year-old female, defaulted three weeks before treatment completion (at 21 months). The girl declined both antiretroviral and anti-TB treatments despite an excellent clinical response and lack of adverse events. We traced the patient and identified a non-supportive, problematic family environment (parental death, history of sexual abuse). Her elder sister, also HIV/MDR-TB co-infected and part of this same cohort, had died. The girl had a partner to whom she was initially unwilling to disclose her status, but eventually did give her consent for couple counseling.

Refusal to start treatment and treatment interruptions were frequently (4/8) observed in this cohort of adolescents. An example illustrating the complexity of treatment acceptance and adherence is an orphan male patient who had started anti-TB treatment but refused to start on antiretroviral treatment. He later abandoned his anti-TB treatment only to restart 5 months later as his condition had deteriorated. He is presently on both treatment regimens. However the family is worried about TB transmission and is trying to send him to a hospice. As a result, the patient is experiencing behavioral issues due to a perceived fear of abandonment. Constant supportive counseling has thus far ensured that he stays on treatment, while additional family counseling is being offered.

All eight patients (100%) who started on treatment experienced adverse events (AE); 2 required permanent discontinuation of the culprit drug and 2 patients had to be hospitalized due to AEs (both for hypokalaemia). The most common adverse events were gastrointestinal intolerance (5/8 patients) followed by peripheral neuropathy (3/8) and psychiatric events (3/8). Adverse events and the time of occurrence are shown in Table 4. None of the eight patients has required permanent discontinuation of the entire second-line anti-TB or antiretroviral regimen.

**Table 4:** Treatment-related adverse events in the Mumbai HIV/MDR-TB co-infected adolescent cohort, 2007–2013.

	Age/Sex	2^ND^ line TB Regimen	ART Regimen	Adverse events (AEs)	Grade	Possible culprit drug	Time (weeks)
1	14/M	Cm,Lfx,Eto,Cs,Amcl	D4T+3TC+EFV	Convulsions	severe	Cs	5
				GI Intolerance	mild	Eto	9
				Peripheral Neuropathy	mild	D4T, Cs	56
2	10/M	Km,Mfx,Eto,Cs,PAS,Z,Amcl	D4T+3TC+NVP	Hearing Loss	mild	Km	4
3	18/F	Km,Mfx,Eto,Cs,PAS	TDF+3TC+EFV (previouslyon d4T)	Peripheral Neuropathy	moderate	D4T	4
4	13/F	No TB Treatment	No ART				
5	18/F	No TB Treatment	No ART				
6	16/F	Cs,Mfx,Eto,PAS,Z,H,Amcl	D4T+3TC+EFV	GI Intolerance	mild	Eto, PAS	1
				Psychiatric (Psychosis)	severe	Cs, EFV	2
7	15/F	Km,Mfx,Eto,Cs,Amcl,H,E	AZT+3TC+NVP	GI Intolerance	moderate	Eto, Amcl	2
				Anxiety Disorder	mild	Cs	2
8	19/F	Cm,Mfx,Eto,Cs,H,Cfz,Z	TDF+3TC+ATV/r	Nephrotoxicity	mild	Cm, TDF	4
				Psychiatric (Psychosis)	severe	Cs	20
9	14/M	Cm,Mfx,Eto,Cs,PAS,Z	ABC+3TC+LPV/r	GI Intolerance	moderate	Eto, PAS	4
				Hypothyroidism	mild	Eto, PAS	12
				Hypokalemia	mild	Cm	40
10	16/M	No TB Treatment	No ART				
11	18/M	Cm,Lfx,Eto,Cs,Amcl,Hh,Cfz	TDF+3TC+EFV	GI Intolerance	mild	Eto, Amcl	1
				Hypokalemia	moderate	Cm	4
				Peripheral Neuropathy	mild	Cs, Eto	9
				Hypothyroidism	mild	Eto	11

M: male; F: female; S: streptomycin; Cm: capreomycin; Km: kanamycin; Lfx: levofloxacin; Eto: ethionamide; CS: cycloserine; Amcl: amoxicillin/clavulanic acid; Mfx: moxifloxacin; PAS: para-aminosalicylic acid; Z: pyrazinamide; H: isoniazid; Hh: high dose isoniazid; E; ethambutol, Cfz: clofazimine; GI: gastrointestinal; CrCl: creatinine clearanc.

## Discussion

This study shows poor treatment outcomes in a cohort of HIV-infected adolescents being treated for MDR-TB. Compared with their adult counterparts in a previous publication [23], these adolescents have both higher rates of death and default. These results are of concern and suggest the need for urgent interventions.

The majority of the adolescents (6/11) in this cohort died, and there may be several explanations for this. First, HIV co-infection has been shown to be a risk factor for mortality among persons with MDR-TB [24]. Co-infected patients may have severe forms of Immune Reconstitution Inflammatory Syndrome (IRIS) following ART initiation that can be associated with mortality [25]. There may be drug-drug interactions between second-line anti-TB medications and ART that could result in decreased efficacy of therapy [26]. Second, extent of disease and drug resistance, particularly to the fluoroquinolones, have also been associated with worse treatment outcomes in other cohorts [27]–[28] and may have played a role here, given that 75% of available DST results showed resistance to this group of drugs.

While these factors may have played a role in the high mortality seen in this study, it should be noted that four of the six patients who died did so either prior to or within three weeks of initiating therapy. Such “early deaths” have been described in other cohorts and are usually considered “failure to treat” as opposed to “treatment failures” [29]. These deaths can generally be ascribed to problems with timely diagnosis and referral rather than ineffective treatment practices [30]. The high early mortality in this cohort suggests that special strategies should be deployed to diagnose and refer adolescents with suspected MDR-TB early in the course of their disease [31]. Although adolescents may be able to more easily provide sputum samples for diagnosis when compared with children, they may be less likely to follow up on referrals [32]. A potential strategy for earlier diagnosis is active screening and immediate clinical referral of all household contacts of persons with MDR-TB [33], given that more than one-third of the adolescents in this study had a known MDR-TB contact; had they been identified through an active case-finding strategy, perhaps they would have been diagnosed more quickly and benefitted from earlier treatment.

Discontinuation of therapy was also frequently reported in this study, with three (37.5%) of eight adolescents starting both antiretroviral and anti-TB therapy eventually defaulting. This is compared with a general loss to follow-up rate in this HIV programme of less than 3% [34]. HIV co-infection may have been a factor in patient discontinuation of therapy due to increased pill burden on ART or the occurrence of adverse events [35]–[37] that may result from interactions between TB and HIV drugs, or additive drug-related side effects. In addition to the complications of HIV co-infection, this adolescent cohort has had other risk factors for default from therapy, including family and social problems, unwillingness to disclose their status, and discrimination.

The story of the adolescent patient who defaulted just a few weeks before treatment completion clearly illustrates the complexity of factors that influence the behavior of adolescents who are HIV-infected and at the same time have to go through a long course of an intolerable and toxic treatment regimen. We have observed that patients that begin to feel better while on treatment prefer to get back to their normal routine: these activities of daily living then take priority over their health. All adolescents in this Mumbai cohort attended school at some point; it is interesting to mention that none of them has disclosed their status at school.

All patients on treatment in this adolescent cohort experienced similar adherence issues: they all reported the high pill burden, the side effects, and the long duration of the treatment as major challenges in their daily life. As expected, patients with good support and patients who experienced low to no discrimination demonstrated better adherence. We have found that supportive counseling is important not just for the patient but also for the family. Education about treatment and adherence issues is important for the family and the caretakers so that they understand the need to support the patient through this difficult treatment. On the other hand, adolescents expressed that “the constant nagging of the caretaker” worked against their adherence. Despite the degree of support and the exposure to stigma and discrimination, all adolescents were conscious of how conspicuous they were during their daily visit to DOT centers, so much so that some preferred to avoid going to the DOT centers at the expense of their health. Adolescents want to experience “normal” lives and “pretended to be normal” rather than “taking the steps to a better health” (patients quotes recorded during routine counseling sessions).

Adverse events were frequent in this cohort and psychiatric events were worryingly common. We have recently introduced a baseline mental health evaluation in order to screen for depression and other mental health issues. We have also found that aggressive management of adverse events is necessary to ensure short and long term adherence.

This study has a number of limitations, most of which are related to the small sample size. Clearly, there is a need to evaluate adolescents undergoing treatment for MDR-TB on a larger scale. Second, all of the patients were infected with HIV and thus the results are not likely generalizable to HIV-negative adolescents with MDR-TB. Finally, this was a retrospective review of medical records that is subject to all the biases inherent with such methodology. In spite of these limitations, the trends identified in this small cohort are of sufficient concern to be reported to the scientific community so that increased attention can be given to adolescent populations with MDR-TB.

### Conclusion

Treatment outcomes seen in HIV-infected adolescents receiving community-based MDR-TB treatment in Mumbai, India were overwhelming poor, with a majority of patients either dying or defaulting from therapy. While some of this may have been due to the complicated treatment regimens required for people with HIV and MDR-TB in general, the data suggest the need for targeted interventions focused on adolescents. Active case-finding, especially among younger contacts of DR-TB cases, and shepherded referrals into treatment could decrease mortality from the disease. Targeted adherence counseling and social support around life events that are common in adolescence could also be a strategy for reducing discontinuation of therapy. Excellent treatment outcomes should be possible for adolescents suffering from MDR-TB, given the encouraging treatment results seen in both pediatric and adult cohorts with the disease. Operational research looking at interventions to improve outcomes in adolescents is sorely needed. Strategies that account for the unique needs of this population should be developed and adapted to ensure a safe and healthy transition into adulthood.
